# Our Associate and Corresponding Editors

**Published:** 1873-01

**Authors:** 


*Our Associate and Corresponding Editors.*

With a view to neatness, and that larger type could be used, we have transferred the names of Associate and Corresponding Editors from the title page to the second of the cover. We hope they will be pleased with the change.

We also take this opportunity to tender our kindest regards to each and all of them. We sincerely thank them for the generous efforts they have made in behalf of "their" journal, and trust they will not relax in their exertions for the Record in the future.

